# Chart of the Physiological Arrangement of Cranial Nerves

**Published:** 1869-07

**Authors:** Edward Rives


### CHART OF THE PHYSIOLOGICAL ARRANGEMENT OF CRANIAL NERVES.

BY EDWARD RIVES, M. D.

This tabular arrangement of Cranial Nerves is a compilation suggested by the difficulty which students encounter in remembering this subject, and also by the frequency with which practitioners find it necessary to refer to their books for facts connected with it. Its object, of course, is simply to refresh the memory at a glance, and suggests details, the explanation of which must be sought for elsewhere.

Printed in large type, on a sheet 28 inches by 15 inches. Price 60 cents, mounted on card board, or folded in cloth case. The latter sent by mail on receipt of the price.

Robert Clarke & Co ,
Publishers, Cincinnati.

